# Cell Lineage Specific Distribution of H3K27 Trimethylation Accumulation in an *In Vitro* Model for Human Implantation

**DOI:** 10.1371/journal.pone.0032701

**Published:** 2012-03-07

**Authors:** Gijs Teklenburg, Charlotte H. E. Weimar, Bart C. J. M. Fauser, Nick Macklon, Niels Geijsen, Cobi J. Heijnen, Susana M. Chuva de Sousa Lopes, Ewart W. Kuijk

**Affiliations:** 1 Department of Reproductive Medicine and Gynaecology, University Medical Center Utrecht, Utrecht, The Netherlands; 2 Laboratory of Neuroimmunology and Developmental Origins of Disease (NIDOD), University Medical Center Utrecht, Utrecht, The Netherlands; 3 Department of Obstetrics and Gynaecology, Division of Developmental Origins of Adult Disease, University of Southampton, Princess Anne Hospital, Southampton, United Kingdom; 4 Hubrecht Institute-KNAW and University Medical Center Utrecht, Utrecht, The Netherlands; 5 Department of Clinical Sciences of Companion Animals, Utrecht University School for Veterinary Medicine, Utrecht, The Netherlands; 6 Department of Anatomy and Embryology, Leiden University Medical Center, Leiden University, Leiden, The Netherlands; National University of Singapore, Singapore

## Abstract

Female mammals inactivate one of their two X-chromosomes to compensate for the difference in gene-dosage with males that have just one X-chromosome. X-chromosome inactivation is initiated by the expression of the non-coding RNA *Xist*, which coats the X-chromosome in cis and triggers gene silencing. In early mouse development the paternal X-chromosome is initially inactivated in all cells of cleavage stage embryos (imprinted X-inactivation) followed by reactivation of the inactivated paternal X-chromosome exclusively in the epiblast precursors of blastocysts, resulting temporarily in the presence of two active X-chromosomes in this specific lineage. Shortly thereafter, epiblast cells randomly inactivate either the maternal or the paternal X-chromosome. XCI is accompanied by the accumulation of histone 3 lysine 27 trimethylation (H3K27me3) marks on the condensed X-chromosome. It is still poorly understood how XCI is regulated during early human development. Here we have investigated lineage development and the distribution of H3K27me3 foci in human embryos derived from an *in-vitro* model for human implantation. In this system, embryos are co-cultured on decidualized endometrial stromal cells up to day 8, which allows the culture period to be extended for an additional two days. We demonstrate that after the co-culture period, the inner cell masses have relatively high cell numbers and that the GATA4-positive hypoblast lineage and OCT4-positive epiblast cell lineage in these embryos have segregated. H3K27me3 foci were observed in ∼25% of the trophectoderm cells and in ∼7.5% of the hypoblast cells, but not in epiblast cells. In contrast with day 8 embryos derived from the co-cultures, foci of H3K27me3 were not observed in embryos at day 5 of development derived from regular IVF-cultures. These findings indicate that the dynamics of H3K27me3 accumulation on the X-chromosome in human development is regulated in a lineage specific fashion.

## Introduction

X-chromosome inactivation (XCI) is a complex process whereby one of the two X-chromosomes in female cells is epigenetically silenced, to obtain similar X-linked gene expression levels as male cells, which have just one X-chromosome. The initiation of X-inactivation depends on RNA from the non-coding gene *Xist*, which coats the X-chromosome in cis and initiates its inactivation [1], [2]. *Xist* recruits the polycomb group complex PRC2 to the X-chromosome that subsequently tri-methylates histone 3 on lysine 27 (H3K27me3), a repressive epigenetic mark that leads to further silencing of the X-chromosome [3]. The presence of H3K27me3 nuclear foci is often used to identify the inactivated X-chromosome in XX female somatic cells.

The activity status of the X-chromosomes in pre- and post-implantation development of female mouse embryos is dynamically regulated. After fertilization, the paternally inherited X-chromosome is inactivated, from the 4-cell stage onward [4]. The paternal X-chromosome remains inactive in the trophectoderm and the primitive endoderm precursors of blastocyst stage mouse embryos (imprinted XCI), but is reactivated in the epiblast precursors of the ICM between E3.5–E4.5, resulting temporarily in two active X-chromosomes (XaXa) [5], [6], [7], [8], [9], [10], [11], [12]. Shortly thereafter in development, one of the active X-chromosomes is randomly inactivated and the resulting pattern of X-chromosome activity is epigenetically transmitted to all daughter cells [10], [11].

In contrast with mouse embryos, *XIST* is initially expressed from both X-chromosomes in female human embryos, indicating that XCI in early development is not conserved between eutherian mammals. [13]. According to Okamoto *et al.*, H3K27me3 is not enriched at the *XIST*-coated chromosome of female human blastocysts [13]. This contrasts with the findings of van den Berg *et al.*, who reported foci of H3K27me3 in ∼30% of the cells of day 6 blastocysts [14]. This discrepancy illustrates that the full dynamics of XCI in human development are still poorly understood. Furthermore, it is unclear whether X-chromosome inactivation is differentially regulated between the different cellular lineages, as it is the case for mouse development.

X-chromosome inactivation and lineage development are difficult to study in humans because, in addition to the restricted availability of human pre-implantation embryos from IVF-cultures, there are technical and ethical obstacles to study human embryos that have initiated implantation in the endometrium. Consequently, the processes of X-chromosome inactivation and lineage development in human embryos beyond the blastocyst stage remain largely elusive.

Culture conditions for embryos derived through in vitro fertilization (IVF) do not favor development beyond day 6 and human implantation sites are inaccessible [15]. We reasoned that using an *in vitro* model for human implantation could overcome these obstacles and open a new window on human development. A system in which human pre-implantation stage embryos are co-cultured with decidualized primary endometrial cells recapitulates a number of processes that are associated with implantation [12], [15], [16]. In this model stromal cells isolated from proliferative endometrium are induced to decidualize by cyclic AMP stimulation, which results in morphological changes reminiscent of the *in vivo* decidual phenotype and the induction of prolactin expression and other products of decidualization [17]. In co-culture, zona pellucida-free pre-implantation embryos attach to, penetrate and ultimately invade through the decidualized endometrial stromal cells. This is accompanied by an increased production of human chorionic gonadotrophin (hCG) by the embryo [12], [16]. We therefore considered this a suitable model system to study the human peri-implantation embryo.

Here, we examined lineage formation and accumulation of H3K27me3 on the X-chromosomes in human day 5 embryos and in human embryos that, from day 5 onwards, have been cultured for an additional 72 h on decidualized endometrial stromal cells. Using immunofluorescence for OCT4 and GATA4 we show that these markers are initially co-expressed in all cells of embryos at day 5 of development, but after 72 h of co-culture expression of OCT4 is restricted to the epiblast, GATA4 expression is restricted to the hypoblast and trophectoderm lineages are OCT4- and GATA4-negative. Female embryos that have been co-cultured on decidualized endometrial cells have distinct H3K27me3 foci, localized to the trophectoderm lineage and to a lesser extend the hypoblast lineages. Interestingly, H3K27me3 foci were not observed in the OCT4-positive pluripotent epiblast cells. We conclude that, in human development, dynamics of H3K27me3 accumulation on the X-chromosome is regulated in a lineage-specific fashion.

## Results

### Development of epiblast and hypoblast precursors in human embryos cultured on decidualized endometrial cells

**Figure 1:**
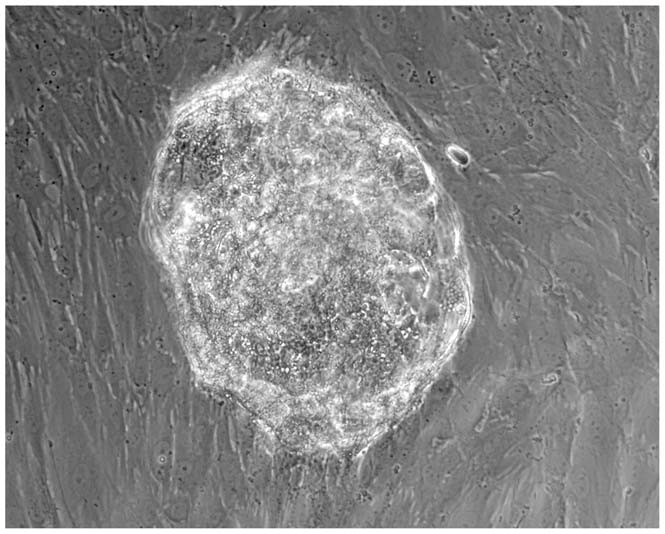
Embryo from co-culture. Human blastocyst attached to decidualized human endometrial stromal cells after 72 h of co-culture.

**Figure 2:**
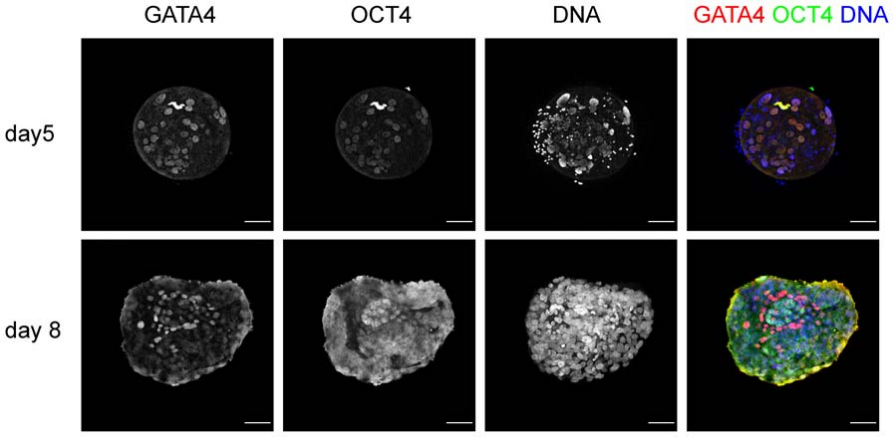
Lineage development in human embryos. Expression of OCT4 and GATA4 in day 5 embryos and in embryos that have been co-cultured with decidualized endometrial cells for 72 h. Scale bars are 50 µm.

Human IVF/ICSI embryos that had been cryopreserved at day 4 were thawed and cultured until day 5. At day 5, embryos were fixed in 4% paraformaldehyde (PFA), or transferred to decidualized endometrial cells and cultured for an additional 72 h (Figure 1). At 72 h of co-culture, human embryos (hereafter referred to as day 8 embryos) were carefully removed and fixed in 4% PFA. Day 5 embryos had on average 31.5 cells (n = 11) and day 8 embryo had on average 414.7 cells (n = 10). To investigate the development of the hypoblast and epiblast lineages in human embryos, we performed immunofluorescence for the transcription factors OCT4 and GATA4 on fixed day 5 embryos and on fixed day 8 embryos. In mouse post-implantation stage embryos, OCT4 is expressed in the epiblast and excluded from the primitive endoderm cells, whereas GATA4 is specifically expressed in the primitive endoderm, but not in the epiblast [18], [19], [20], [21], [22], [23], [24], [25]. In human development, OCT4 is initially expressed in all cells at the early blastocyst stage [26], [27]. In older blastocysts, OCT4 expression is downregulated in the trophectoderm lineage, while expression in the ICM remains high [27],

All nuclei of day 5 embryos were positive for OCT4 (in 8 out of 8 embryos)and GATA4 (in 3 out of 3 embryos) (Figure 2). However, in embryos cultured on decidualized endometrial cells for 72 h, double-immunofluorescence demonstrated the presence of an OCT4-positive cell population covered by a layer of GATA4-positive hypoblast precursors. There were no cells observed that strongly co-expressed GATA4 and OCT4, although weak expression of GATA4 was observed in a few OCT4-positive cells (data not shown). In some day 8 embryos, we also observed OCT4 expression in the cytoplasm of the trophectoderm cells. In day 6 embryos, OCT4 is known to be expressed in both the trophectoderm and the ICM [27]. Cytoplasmic OCT4 staining in the trophectoderm possibly reflects a stage just before its expression is fully downregulated in this lineage.

**Figure 3:**
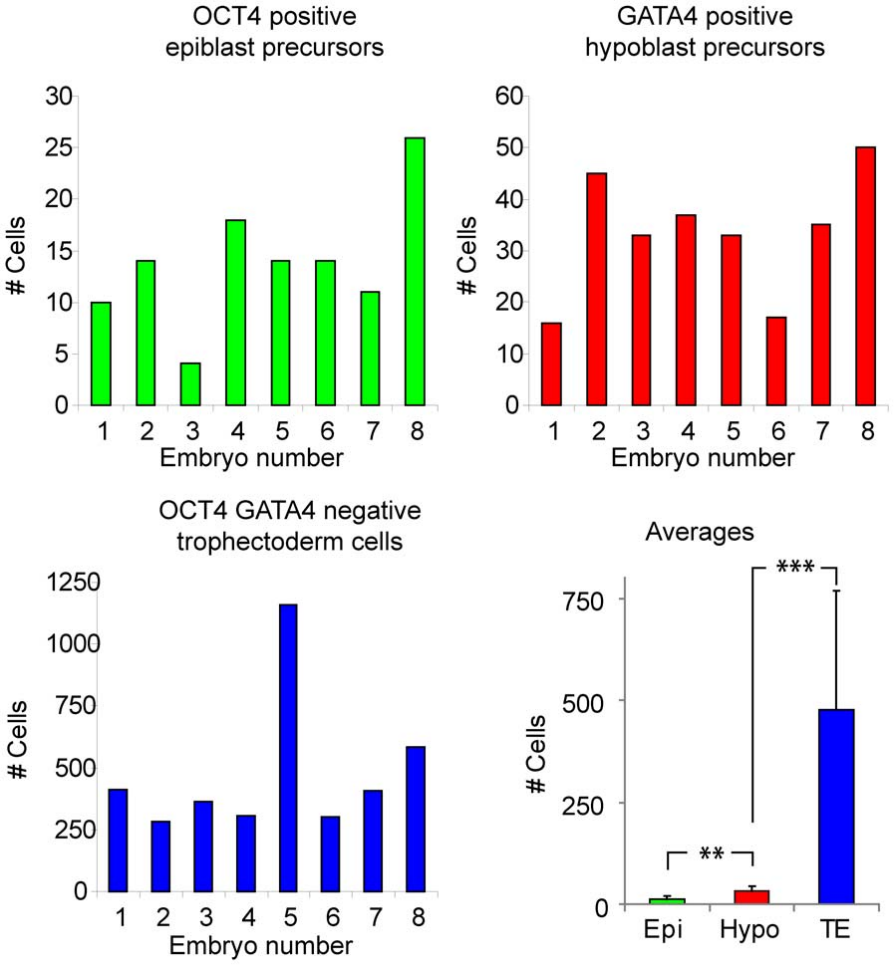
The number of cells per lineage in human embryos. The number of cells in each lineage in embryos that have been co-cultured with decidualized endometrial cells for 72 h. Green bars are the number of OCT4 positive epiblast precursors, red bars are the number of GATA4 positive hypoblast precursors, blue bars are the number of OCT4- and GATA4- negative trophectoderm cells. Error bars denote standard deviation; asterisks denote significant differences: **: p<0.01; ***: p<0.001. Epi = Epiblast lineage; Hypo = Hypoblast lineage; TE = Trophectoderm lineage.

The results above indicate that the strong OCT4-positive cells and strong GATA4-postive cells represent two separate and segregated lineages at day 8 of development. Quantification of the OCT4 positive cells and GATA4 positive cells demonstrated that there were significantly less OCT4 positive epiblast precursors than GATA4 positive hypoblast cells (p<0.01). The majority of cells were negative for OCT4 and GATA4 and based on their outside localization we concluded these were presumptive trophectoderm cells (Figure 3). According to a recently published study, good quality human embryos at day 6 of development have on average 18.6 ICM cells [28]. This is close to our own observation of the number of ICM cells in day 6 human embryos (data not shown). The ICM of day 8 embryos had on average 47.13±16.63 cells (n = 8). Thus human embryos cultured on decidualized endometrial cells from day 6 until day 8 of development show segregation of hypoblast precursors from epiblast precursors and an increase in the absolute number of ICM cells compared to embryos at day 6 of development. These data demonstrate that the co-culture system allows human embryos to progress to a further stage in development than is generally possible under regular culture conditions for IVF embryos. Importantly, our results indicate that co-staining of day 8 embryos with antibodies against OCT4 and GATA4 allows identification of the epiblast, hypoblast and trophectoderm lineages.

### Distribution of H3K27me3 foci in early blastocysts and in post-implantation stage embryos

**Figure 4:**
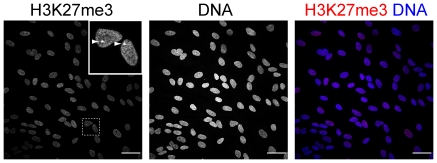
H3K27me3 staining of endometrial stromal cells. Accumulation of H3K27me3 on X-chromosomes was observed in >90% of the cells. Scale bars are 50 µm.

Female cells that carry an inactive X-chromosome can be recognized by the presence of a prominent focus of nuclear H3K27me3. We were able to detect prominent foci of H3K27me3 in >90% endometrial cells using immunofluorescence with an antibody against H3K27me3 (Figure 4). With the same staining we were unable to detect prominent single foci of H3K27me3 in cells of day 5 embryos (n = 8).

**Figure 5:**
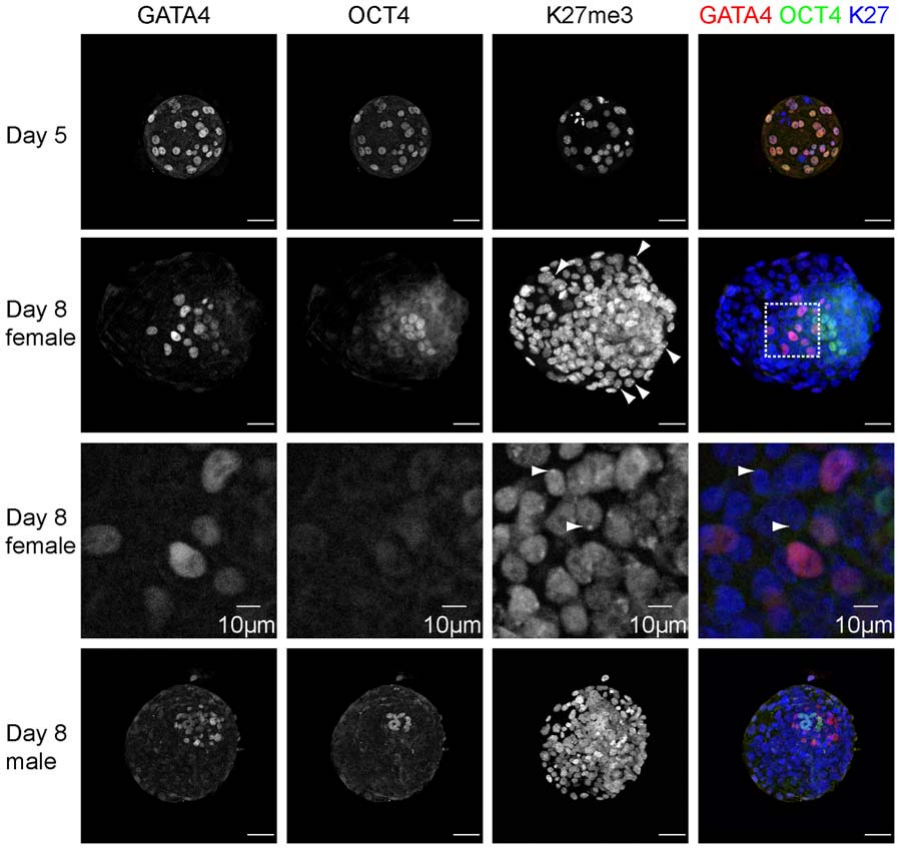
Lineage development and H3K27me3-accumulation on the X-chromosome in human embryos. Expression of GATA4, OCT4, and H3K27me3 in human day 5 pre-implantation and embryos that have been co-cultured with decidualized endometrial cells for 72 h (day 8). Foci of H3K27me3 are diagnostic for an inactivated X-chromosome. Arrowheads denote H3K27me3 foci. The third row panels are enlargements of the indicated area in the merge panel in the above row. Scale bars are 50 µm unless denoted otherwise.

**Figure 6:**
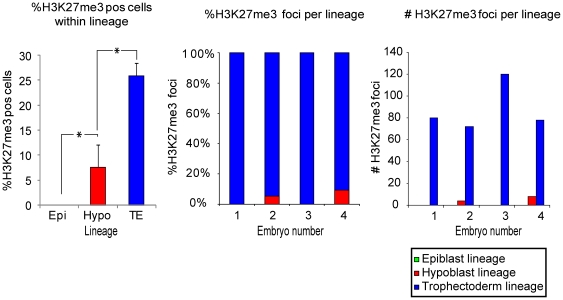
Degree of H3K27me3 foci per lineage. The percentages of cells in each lineage that carry a distinct H3K27me3 focus in embryos that have been co-cultured with decidualized endometrial cells for 72 h. Left panel: relative number of H3K27me3 per lineage as a function of the total number of cells within that lineage; middle panel: absolute values of H3K27me3 foci counted within each lineage for each embryo; right panel: relative number of H3K27me3 foci in each lineage as a function of the total number of foci observed in that embryo. H3K27me3 foci did not reveal any preferential distribution within the embryo. Green: H3K27me3 foci in OCT4 positive epiblast precursors; red: H3K27me3 foci in GATA4 positive hypoblast precursors; blue: H3K27me3 foci in OCT4- and GATA4- negative trophectoderm cells. Asterisks denote significant differences: *p<0.05. Epi = Epiblast lineage; Hypo = Hypoblast lineage; TE = Trophectoderm lineage.

**Figure 7:**
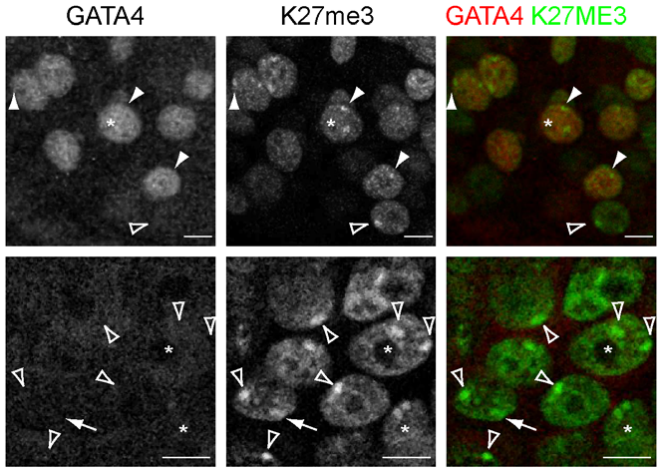
H3K27me3 accumulation in trophectoderm and hypoblast precursors. Expression of GATA4, and H3K27me3 in human embryos that have been co-cultured with decidualized endometrial cells for 72 h. Closed arrowheads denote GATA4 positive cells with H3K27me3 foci. Open arrowheads denote trophectoderm cells negative for OCT4 and GATA4 with distinct H3K27me3 foci. The asterisks mark cells with two H3K27me3 foci. Arrows mark the location of a diffuse H3K27me3 domain. Scale bars are 10 µm.

By contrast, in 4 of the day 8 embryos (n = 8), prominent single H3K27me3 foci, were observed, reminiscent of an inactivated X-chromosome in these cells (Figure 5). In the other 50% of the day 8 embryos no H3K27me3 foci were observed. H3K27me3 foci were not observed in all cells. To determine which cell types carried H3K27me3 foci, we examined if their distribution co-localized with OCT4 or GATA4 positive cells. Many H3K27me3 foci (87.5±21.9 per embryo) were observed in GATA4 and OCT4 negative (and therefore presumably trophectoderm) cells (Figure 6). Nevertheless, a large fraction (∼75%) of the trophectoderm cells did not have clear H3K27me3 foci (Figure 6). Some H3K27me3 foci were also observed in GATA4 positive hypoblast precursors (Figure 6, 7), although significantly less than in the trophectoderm. In a small proportion of cells two foci were observed per cell, in both the trophectoderm and the hypoblast lineages (Figure 7). In the hypoblast and trophectoderm lineages, H3K27me3 domains were also observed that were too diffuse to count as bona fide foci of H3K27me3 (Figure 7). Nevertheless, these diffuse domains might indicate either X-chromosomes that are less compacted than fully inactivated X-chromosomes. Alternatively, these could be areas of (facultative) heterochromatin or repressive chromatin domains.

**Figure 8:**
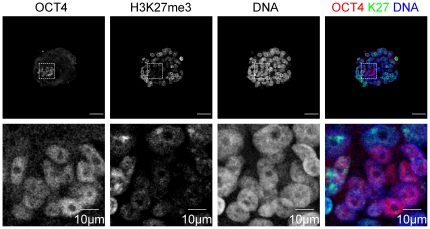
No accumulation of H3K27me3 on the X-chromosomes of epiblast precursors. Expression of OCT4, and H3K27me3 in human embryos that have been co-cultured with decidualized endometrial cells for 72 h. The four bottom panels are an enlargement of the indicated area in the panels directly above. Scale bars are 50 µm unless otherwise indicated.

We investigated the presence of H3K27me3 foci in epiblast precursors. In the OCT4 positive cells, no H3K27me3 foci were observed (Figure 6, 8). These findings indicate that, in the OCT4 positive pluripotent cell population of human embryos, the accumulation of H3K27me3 on the X-chromosome does not follow the same dynamics as in the hypoblast or the trophectoderm cells.

## Discussion

In this study, we have co-cultured of human embryos with decidualized endometrium cells. This system supports the embryo to progress development to a later stage than would be possible in conventional culture systems [15]. In the present study, we demonstrate that human embryos that have been cultured on decidualized endometrial cells have relatively large ICMs in which the epiblast precursors have segregated from the hypoblast precursors. The possibility to extend development of *in vitro*-cultured human embryos enabled us to study the accumulation of H3K27me3 (presumably on the inactive X-chromosome) in the trophectoderm, hypoblast and epiblast cell lineages of human peri-implantation stage embryos. Our findings indicate that X-chromosome-wide accumulation of H3K27me3 in human embryos is regulated in a lineage-specific manner during peri-implantation.

The accumulation of H3K27me3 on the presumably silent X-chromosome was markedly different in embryonic versus extra-embryonic lineages of implanting human day 8 embryos. The trophectoderm contained the highest number of cells with clear H3K27me3 foci, followed by the hypoblast precursors. In a few cells of the hypoblast and the trophectoderm lineages, 2 foci of H3K27me3 could be identified (Figure 7). The presence of two H3K27me3 foci in one cell has also been described for rabbit embryos [13]. H3K27me3 foci were not observed in the OCT4 positive epiblast precursors. This indicates that human embryos have lineage specific dynamics ofH3K27me3 accumulationon the X-chromosome.

We observed expression of OCT4 in all blastomeres of day 5 embryos. Interestingly, OCT4 is initially also expressed in all cells of human blastocysts [26], [27], after which it is first downregulated in the trophectoderm lineage [27], followed by exclusion from the hypoblast lineage and restricted expression in the epiblast lineage during peri-implantation (this study). The current study suggests that there is a lineage specific degree of H3K27me3 accumulation on the X-chromosome, which might be a reflection of the lineage specific loss of OCT-4 expression.

Finally, if the H3K27me3 foci we observed in the current study represent fully silenced X-chromosomes, our findings indicate that in the majority of the cells of human embryos, silencing of the X-chromosome may occur after the embryo has implanted. In the mouse, XCI is regulated by pluripotency factors [29], [30]. The slow onset of H3K27me3 accumulation on the X-chromosomes in human development could be the consequence of the ubiquitous co-expression of pluripotency factors at relative late stages of pre-implantation development. In a similar line of reasoning, the lineage-specific degree of H3K27me3 accumulation in human peri-implantation stage embryos might reflect the lineage specific loss of OCT4 expression.

## Materials and Methods

### Ethics statement and patient selection

This study was approved by the Central Committee on Research Involving Human Subjects (CCMO) in The Netherlands (NL 12481.000.06) and by the local Medical Review Ethics Committee of the University Medical Center in Utrecht. Written informed consent was obtained from all participating subjects for confirmation that supernumerary cryopreserved embryos or endometrial samples could be used for research purposes.

### Embryo collection and co-culture

Ovarian hyperstimulation, oocyte maturation triggering and in vitro fertilization (IVF) or intracytoplasmic sperm injection (ICSI) were performed according to standard clinical protocols as described earlier [31]. Supernumerary, good quality day 4 embryos were cryopreserved as previously described [32]. For this study, embryos were thawed, taken through consecutive washes of 1.25, 1.00, 0.75 and 0.375 mol/l DMSO for 5 minutes each, then transferred to Human Tubal Fluid culture medium supplemented with 10% GPO (human plasma solution; CLB, The Netherlands), overlaid with 1 ml of light paraffin oil (Irvine Scientific, Santa Ana, USA), and cultured at 37°C until day 5 of development under atmospheric oxygen levels and 5% CO_2_. Thirty-nine embryos from 17 patients survived the thawing procedure and extended culture period. 14 embryos were subjected to 0.1% Pronase/10% GPO treatment to remove the zona pellucida, before they were transferred to decidualized endometrial cells. For the co-culture experiments, primary endometrial stromal cells were purified, as previously described [33], from a single proliferative phase biopsy sample obtained from a patient with no uterine pathology or a history of recurrent pregnancy loss. Endometrial stromal cells were seeded into 16 mm wells (0.5×10^5^ cells per well) in DMEM/F12 complete medium and grown until confluence. Decidualization was induced by the addition of 0.5 mM of 8-bromoadenosine 3′,5′-cyclic monophosphate (8-Br-cAMP; Sigma, UK) in combination with 1 µM medroxyprogesterone acetate (MPA; Sigma). This medium was changed every 48 hours. Individual blastocysts were then seeded onto a confluent layer of endometrial cells that had been decidualized for 5 days. Co-cultures were maintained in DMEM/F-12 complete medium for 72 hours at 37°C under atmospheric oxygen levels and 5% CO_2_. At the end of the co-culture period, the embryos were carefully removed from the stromal cells with a mouth pipet.

### Immunofluorescence

Day 5 embryos were washed in PBS and fixed for 10 minutes at room temperature (RT) in 4% PFA. Day 8 human blastocysts were carefully detached from the decidualized endometrium cells, washed in PBS and fixed for 10 minutes at room temperature (RT) in 4% PFA. The rest of the staining procedure was similar for day 5 and day 8 embryos. Embryos were washed briefly in PBS with 10% Foetal Calf Serum (FCS) and 0.1% triton x-100 (PBST). Subsequently, embryos were permeabilized in PBS with 10%FCS and 0.5% triton x-100 for 15–30 minutes at RT. Embryos were incubated in PBST for 1 hour at RT followed by overnight incubation at 4°C in primary antibodies diluted in PBST. The primary antibodies used were: rabbit anti-H3K27me3 (Millipore, Temecula, USA), goat anti-OCT4 (Santa Cruz Biotechnology, Santa Cruz, USA), and mouse anti-GATA4 (Santa Cruz) diluted in blocking solution. The embryos were then washed in PBST and transferred to PBST containing Alexa fluor conjugated secondary antibodies (Molecular probes, Invitrogen, Venlo, the Netherlands). In the case of triple labeling, embryos were first incubated in PBST with Alexa633 donkey anti-goat, before they were transferred to PBST containing goat anti-rabbit and goat-anti mouse secondary antibodies. After 1 hour in secondary antibody solution, embryos were washed and mounted in Vectashield mounting medium containing DAPI (Brunschwig Chemie, Amsterdam, The Netherlands). Fluorescent signals were visualized using a Leica SPE confocal laser scanning microscope (Leica, Rijswijk, the Netherlands) and acquired images were analyzed with ImageJ [34]. We defined bright non-diffuse signals as foci of H3K27me3.A Student's two-sided t-test was used to evaluate statistical differences. A difference was determined to be significant when P<0.05.
